# Supplementary material for: Unraveling Heterogeneity of Coral Microbiome Assemblages in Tropical and Subtropical Corals in the South China Sea
Source: Microorganisms. 2020 Apr 21;8(4):604. doi: 10.3390/microorganisms8040604 (PMC7232356; doi:10.3390/microorganisms8040604)
Supplement: Supplementary file 1 [file microorganisms-08-00604-s001.zip › microorganisms-760544-supplementary/Additional file 2-Table S1-S3.docx]

**Table S1.** Environmental data during sampling period (mean ± sd).

| Reef regions | Temperature (°C) | Depth  (m) | Salinity | NO_3_^-^  (µg/L) | NO_2_^-^  (µg/L) | NH_4_^+^  (µg/L) | PO_4_^3-^(µg/L) |
| --- | --- | --- | --- | --- | --- | --- | --- |
| Sanya Bay (SY) | 31.44 ±  0.30 | 2.94 ±  0.16 | 33.70 ± 0.17 | 73.06 ± 1.09 | 3.27 ± 0.16 | 16.47 ±  0.34 | 5.40 ±  0.21 |
| Daya Bay (DY) | 24.24±  0.30 | 3.03±  0.27 | 33.67 ±  0.19 | 29.25 ±  0.34 | 3.28 ± 0.15 | 10.39 ±  0.29 | 2.27 ±  0.28 |

**Table S2.** Summary of NGS data and goods_coverage. Including sampled corals, number of 16S rRNA sequences and Q20, number of OTUs, Shannon Diversity(Shannon_H) and goods_coverage.

| **Corals** | **Q20(%)-R1** | **Q20(%)-R2** | **sequences** | **Number of OTUs** | **Shannon Diversity** | **goods_coverage** |
| --- | --- | --- | --- | --- | --- | --- |
| *A. sp.* SY1 | 92 | 92 | 38919 | 237 | 3.6 | 1 |
| *A. sp.* SY2 | 92 | 92 | 42055 | 296 | 3.6 | 1 |
| *A. sp.* SY3 | 93 | 93 | 73252 | 343 | 3.9 | 1 |
| *A. sp.* SY4 | 92 | 92 | 67653 | 269 | 4.0 | 1 |
| *A. sp.* SY5 | 92 | 92 | 49761 | 424 | 3.1 | 1 |
| *A. sp.* SY6 | 93 | 93 | 84582 | 306 | 2.4 | 1 |
| *A. sp.* SY7 | 92 | 92 | 44872 | 323 | 2.6 | 1 |
| *A. sp.* SY8 | 92 | 92 | 47448 | 315 | 2.3 | 1 |
| *A. sp.* SY9 | 93 | 93 | 32623 | 413 | 3.0 | 1 |
| *A. sp.* DY1 | 92 | 92 | 78063 | 591 | 4.3 | 1 |
| *A. sp.* DY2 | 92 | 92 | 87545 | 490 | 3.9 | 1 |
| *A. sp.* DY3 | 93 | 93 | 36682 | 544 | 3.9 | 1 |
| *A. sp.* DY4 | 92 | 92 | 78198 | 575 | 4.1 | 1 |
| *A. sp.* DY5 | 92 | 92 | 57545 | 540 | 4.2 | 1 |
| *A. sp.* DY6 | 93 | 93 | 99747 | 768 | 3.8 | 1 |
| *A. sp.* DY7 | 92 | 92 | 94329 | 498 | 3.9 | 1 |
| *A. sp.* DY8 | 92 | 92 | 108286 | 960 | 5.4 | 0.996 |
| *A. sp.* DY9 | 93 | 93 | 107926 | 550 | 3.5 | 1 |
| *A. sp.* DY10 | 92 | 92 | 87512 | 586 | 3.9 | 1 |
| *G. fascicularis* SY1 | 92 | 92 | 49476 | 625 | 4.2 | 1 |
| *G. fascicularis* SY2 | 93 | 93 | 57877 | 725 | 4.2 | 1 |
| *G. fascicularis* SY3 | 92 | 92 | 88815 | 578 | 4.4 | 1 |
| *G. fascicularis* SY4 | 92 | 92 | 45832 | 429 | 4.2 | 1 |
| *G. fascicularis* SY5 | 93 | 93 | 95633 | 585 | 4.1 | 1 |
| *G. fascicularis* DY1 | 91 | 91 | 82789 | 869 | 4.6 | 1 |
| *G. fascicularis* DY2 | 93 | 93 | 63903 | 591 | 4.3 | 1 |
| *G. fascicularis* DY3 | 92 | 92 | 79490 | 917 | 3.9 | 1 |
| *G. fascicularis* DY4 | 93 | 93 | 46429 | 674 | 4.2 | 1 |
| *G. fascicularis* DY5 | 91 | 91 | 78760 | 673 | 4.6 | 1 |
| *G. fascicularis* DY6 | 93 | 93 | 85829 | 618 | 4.3 | 1 |
| *G. fascicularis* DY7 | 92 | 92 | 75229 | 551 | 4.1 | 1 |
| *G. fascicularis* DY8 | 93 | 93 | 63316 | 488 | 4.3 | 1 |
| *G. fascicularis* DY9 | 91 | 91 | 59351 | 730 | 4.1 | 1 |
| *P. lamellina* SY1 | 93 | 93 | 90131 | 1240 | 4.3 | 0.997 |
| *P. lamellina* SY2 | 92 | 92 | 79584 | 495 | 4.9 | 1 |
| *P. lamellina* SY3 | 93 | 93 | 83144 | 593 | 3.0 | 1 |
| *P. lamellina* SY4 | 91 | 91 | 64523 | 651 | 3.1 | 1 |
| *P. lamellina* SY5 | 93 | 93 | 105042 | 905 | 4.2 | 1 |
| *P. lamellina* DY1 | 92 | 92 | 73389 | 688 | 3.4 | 1 |
| *P. lamellina* DY2 | 93 | 93 | 66797 | 678 | 4.3 | 1 |
| *P. lamellina* DY3 | 91 | 91 | 60289 | 977 | 4.2 | 1 |
| *P. lamellina* DY4 | 93 | 93 | 60559 | 666 | 4.1 | 1 |
| *P. lutea* SY1 | 92 | 92 | 64880 | 1087 | 4.1 | 0.997 |
| *P. lutea* SY2 | 93 | 93 | 70250 | 946 | 5.0 | 1 |
| *P. lutea* SY3 | 91 | 91 | 70455 | 1309 | 4.8 | 1 |
| *P. lutea* SY4 | 93 | 93 | 14827 | 398 | 5.7 | 1 |
| *P. lutea* DY1 | 92 | 92 | 11242 | 430 | 4.7 | 1 |
| *P. lutea* DY2 | 93 | 93 | 74431 | 633 | 5.0 | 1 |
| *P. lutea* DY3 | 91 | 91 | 55292 | 956 | 4.4 | 1 |
| *P. lutea* DY4 | 93 | 93 | 43953 | 879 | 4.8 | 1 |
| *P. lutea* DY5 | 92 | 92 | 54355 | 692 | 4.9 | 1 |
| *F. speciosa* SY1 | 93 | 93 | 49495 | 722 | 3.5 | 1 |
| *F. speciosa* SY2 | 91 | 91 | 94650 | 1432 | 4.1 | 1 |
| *F. speciosa* SY3 | 93 | 93 | 69697 | 603 | 5.5 | 1 |
| *F. speciosa* SY4 | 92 | 92 | 83943 | 1363 | 4.5 | 1 |
| *F. speciosa* SY5 | 93 | 93 | 55250 | 506 | 5.4 | 1 |
| *F. speciosa* SY6 | 91 | 91 | 67368 | 931 | 4.3 | 1 |
| *F. speciosa* SY7 | 93 | 93 | 66939 | 562 | 5.0 | 1 |
| *F. speciosa* DY1 | 92 | 92 | 73296 | 583 | 4.2 | 1 |
| *F. speciosa* DY2 | 93 | 93 | 49331 | 836 | 4.1 | 1 |
| *F. speciosa* DY3 | 91 | 91 | 55386 | 741 | 5.0 | 1 |
| *F. speciosa* DY4 | 93 | 93 | 57824 | 740 | 4.3 | 1 |
| *F. speciosa* DY4 | 92 | 92 | 50773 | 1215 | 4.4 | 0.991 |
| *S. glaucum* SY1 | 93 | 93 | 74176 | 270 | 1.1 | 1 |
| *S. glaucum* SY2 | 91 | 91 | 58160 | 225 | 2.3 | 1 |
| *S. glaucum* SY3 | 93 | 93 | 71322 | 304 | 1.1 | 1 |
| *S. glaucum* SY4 | 92 | 92 | 122393 | 455 | 1.2 | 1 |
| *S. glaucum* SY5 | 93 | 93 | 84640 | 429 | 1.6 | 1 |
| *S. glaucum* DY1 | 91 | 91 | 44283 | 380 | 3.0 | 1 |
| *S. glaucum* DY2 | 93 | 93 | 40297 | 339 | 2.7 | 1 |
| *S. glaucum* DY3 | 92 | 92 | 38786 | 389 | 3.1 | 1 |
| *S. glaucum* DY4 | 93 | 93 | 30047 | 471 | 3.3 | 1 |
| *S. glaucum* DY5 | 91 | 91 | 27617 | 275 | 3.0 | 1 |
| Total (73 ) |  |  | 4798543 | 7604 |  |  |
| Seawater SY |  |  | 94516 | 952 | 4.8 | 1 |
| Seawater DY |  |  | 95907 | 503 | 3.6 | 1 |
| SY_VS_DY |  |  |  |  | 5.4/5.0 |  |

**Table S3.** Significance tests of coral microbiome across different coral samples, respectively. Permutational multivariate analysis of variance (PERMANOVA) based on Bray-Curtis distance matrices was used.

| Group | R | P |
| --- | --- | --- |
| *Acropora* sp. (SY) vs *Acropora* sp. (DY) | 0.6598 | 0.001 |
| *G. fascicularis* (SY) vs *G. fascicularis* (DY) | 1 | 0.001 |
| *P. lamellina* (SY) vs *P. lamellina* (DY) | 0.9062 | 0.012 |
| *P. lutea* (SY) vs *P. lutea* (DY) | 0.1625 | 0.142 |
| *F. speciosa* (SY) vs *F. speciosa* (DY) | 1 | 0.002 |
| *S. glaucum* (SY) vs *S. glaucum* (DY) | 0.984 | 0.011 |
| *Acropora* sp. (SY) vs *G. fascicularis* (SY) | 1 | 0.001 |
| *Acropora* sp. (SY) vs *P. lamellina* (SY) | 1 | 0.002 |
| *Acropora* sp. (SY) vs *P. lutea* (SY) | 1 | 0.002 |
| *Acropora* sp. (SY) vs *F. speciosa* (SY) | 1 | 0.002 |
| *Acropora* sp. (SY) vs *S. glaucum* (SY) | 1 | 0.001 |
| *G. fascicularis* (SY) vs *P. lamellina* (SY) | 0.988 | 0.012 |
| *G. fascicularis* (SY) vs *P. lutea* (SY) | 1 | 0.007 |
| *G. fascicularis* (SY) vs *F. speciosa* (SY) | 0.9963 | 0.003 |
| *G. fascicularis* (SY) vs *S. glaucum* (SY) | 1 | 0.005 |
| *P. lamellina* (SY) vs *P. lutea* (SY) | 1 | 0.005 |
| *P. lamellina* (SY) vs *F. speciosa* (SY) | 0.9668 | 0.003 |
| *P. lamellina* (SY) vs *S. glaucum* (SY) | 0.992 | 0.008 |
| *P. lutea* (SY) vs *F. speciosa* (SY) | 0.992 | 0.008 |
| *P. lutea* (SY) vs *S. glaucum* (SY) | 0.9875 | 0.005 |
| *F. speciosa* (SY) vs *S. glaucum* (SY) | 1 | 0.003 |
| *Acropora* sp. (DY) vs *G. fascicularis* (DY) | 0.1682 | 0.019 |
| *Acropora* sp. (DY) vs *P. lamellina* (DY) | 0.2882 | 0.065 |
| *Acropora* sp. (DY) vs *P. lutea* (DY) | 0.4044 | 0.019 |
| *Acropora* sp. (DY) vs *F. speciosa* (DY) | 0.03273 | 0.307 |
| *Acropora* sp. (DY) vs *S. glaucum* (DY) | 0.8335 | 0.001 |
| *G. fascicularis* (DY) vs *P. lamellina* (DY) | 0.9365 | 0.002 |
| *G. fascicularis* (DY) vs *P. lutea* (DY) | 0.6116 | 0.002 |
| *G. fascicularis* (DY) vs *F. speciosa* (DY) | 0.2889 | 0.054 |
| *G. fascicularis* (DY) vs *S. glaucum* (DY) | 1 | 0.001 |
| *P. lamellina* (DY) vs *P. lutea* (DY) | 0.25 | 0.055 |
| *P. lamellina* (DY) vs *F. speciosa* (DY) | 1 | 0.008 |
| *P. lamellina* (DY) vs *S. glaucum* (DY) | 1 | 0.008 |
| *P. lutea* (DY) vs *F. speciosa* (DY) | 0.208 | 0.032 |
| *P. lutea* (DY) vs *S. glaucum* (DY) | 0.932 | 0.01 |
| *F. speciosa* (DY) vs *S. glaucum* (DY) | 1 | 0.008 |
| Corals vs Seawater | 0.5972 | 0.015 |
| Corals (SY) vs Corals (DY) | 0.3231 | 0.001 |
| Whole | 0.8026 | 0.001 |
