# Supplementary material for: Unraveling Heterogeneity of Coral Microbiome Assemblages in Tropical and Subtropical Corals in the South China Sea
Source: Microorganisms. 2020 Apr 21;8(4):604. doi: 10.3390/microorganisms8040604 (PMC7232356; doi:10.3390/microorganisms8040604)

**Figure S1.** Bar plot of the value of environmental factors in the tropical SY and subtropical DY reefs in the SCS. The value of environmental factors is mean ± SE. Stars above bar plots denote statistically significant differences between tropical SY and subtropical DY (*P*<0.001).

**
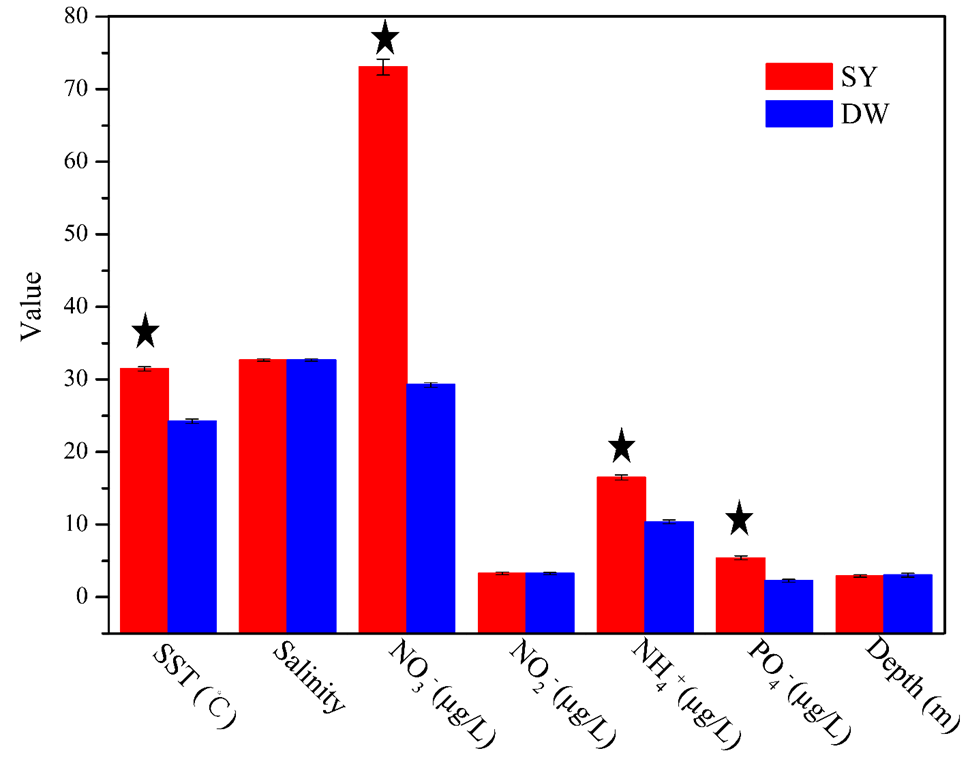
**

**Figure S2.** Bar plot of the relative abundance of coral microbiome at family level (top 30 family). Each bar represents the relative abundance of different bacteria phylum in one coral sample or fiters of seawater sample.


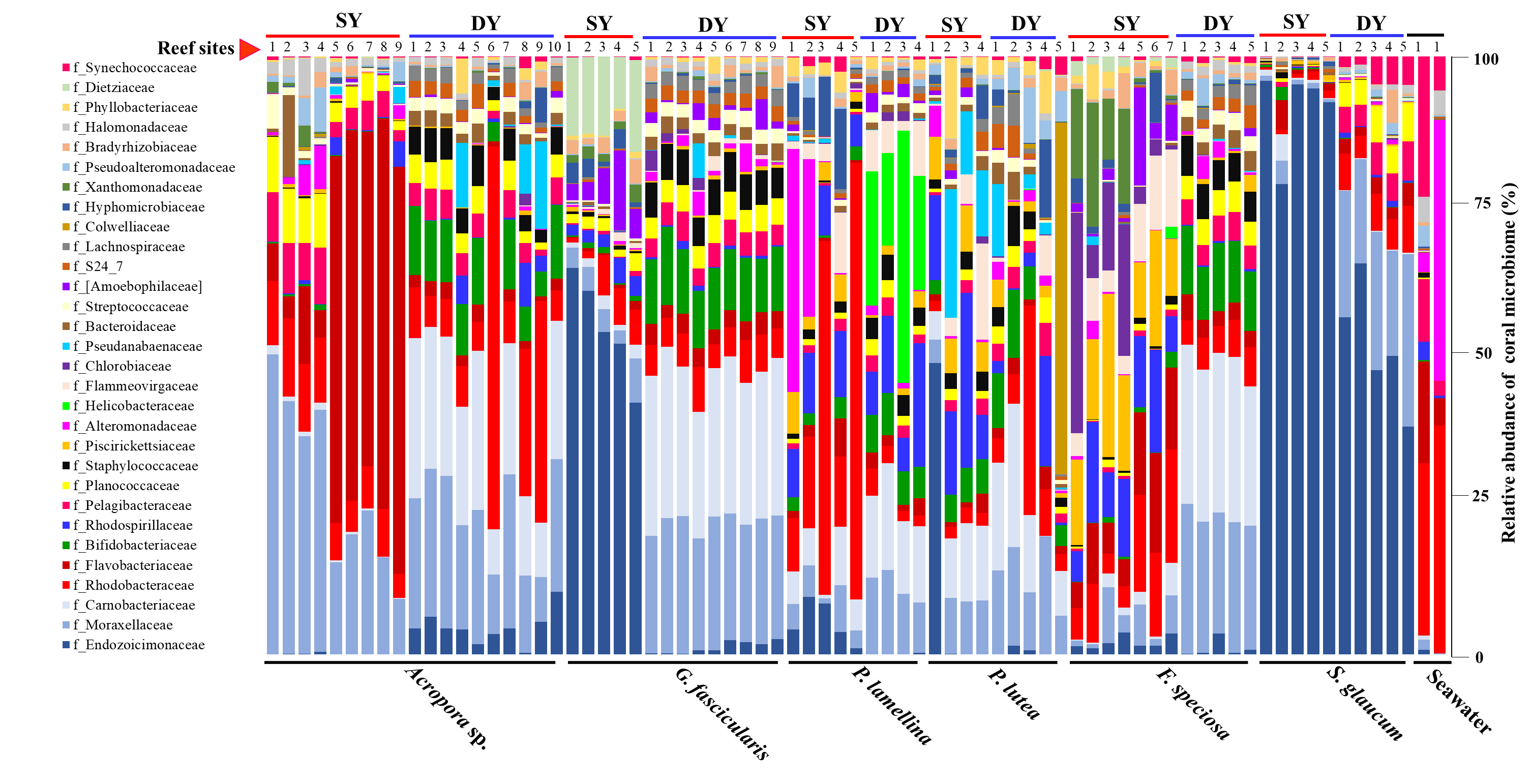

Supplement: Supplementary file 1 [file microorganisms-08-00604-s001.zip › microorganisms-760544-supplementary/Additional file 3-Figure S1-S2.docx]
